# Strong expression of ID1 protein is associated with decreased survival, increased expression of ephrin-A1/EPHA2, and reduced thrombospondin-1 in malignant melanoma

**DOI:** 10.1038/sj.bjc.6602792

**Published:** 2005-09-27

**Authors:** O Straume, L A Akslen

**Affiliations:** 1 The Gade Institute, Section for Pathology, University of Bergen, Haukeland University Hospital, Bergen, Norway

**Keywords:** ID1, ETS, melanoma, p16, Tsp-1, ephrin-A1, prognosis

## Abstract

The ID1 protein, an inhibitor of basic helix–loop–helix transcription factors, has been involved in multiple cellular processes including cell cycle regulation, apoptosis, and angiogenesis. To evaluate the importance of ID1 in malignant melanoma, tumour cell expression was examined by immunohistochemistry in 119 cases of nodular melanoma using tissue microarray technique, and related to multiple tumour markers including proliferation, p16 expression, angiogenesis and patient survival. Strong ID1 expression was significantly associated with increased tumour thickness, and significantly reduced survival. Also, increased ID1 was associated with loss of thrombospondin-1 (TSP-1) expression, a known inhibitor of angiogenesis, and increased intensity of ephrin-A1 and its receptor EPHA2. Presence of *BRAF* mutations was related to strong ID1 expression, but there was no relationship with p16 protein expression. Further, no significant correlation was found between ID1 and microvessel density. In conclusion, our study supports a significant role of the ID1 protein in melanoma progression and patient prognosis. The absence of correlation with p16 protein expression and angiogenesis suggests that other regulatory pathways and mechanisms might be influenced by ID1 in melanomas. An inverse relation between ID1 and TSP-1 expression support an important role of ID1 in the regulation of this complex multitarget protein.

Several molecular pathways are important for the development and progression of cutaneous melanoma (Herlyn *et al*, 2000; Bennett and Medrano, 2002) (Keller-Melchior-98, Pavey-02), and we have previously reported that both tumour cell proliferation and angiogenesis are increased in aggressive tumour subgroups (Straume *et al*, 2000; Straume and Akslen, 2001). Especially, deregulation of the *CDKN2A/p16* pathway was apparent with loss of p16 protein expression in 45% of the cases (Straume *et al*, 2000; Straume and Akslen, 2001), although the mechanisms are not clear. Lack of p16 expression was associated with increased tumour cell proliferation by Ki-67 expression and reduced survival in melanomas. In addition, we found a strong prognostic impact of angiogenesis as estimated by microvessel density (MVD), as well as significant influence of thrombospondin-1 (TSP-1) staining in the tumour stroma (Straume and Akslen, 2001). The purpose of our present study was to examine the possible regulatory role of ID1 and ETS transcription factors in melanoma progression, especially since they are known to influence both the *CDKN2A/p16* pathway and angiogenesis regulation (Yates *et al*, 1999; Alani *et al*, 2001).

The family of Id proteins consists of four members capable of inhibiting basic helix–loop–helix transcription factors (Norton *et al*, 1998). Recent studies have implicated a regulatory role of Id proteins in multiple processes such as cell cycle progression (Lasorella *et al*, 1996; Israel *et al*, 1999), apoptosis (Norton and Atherton, 1998), and angiogenesis (Lyden *et al*, 1999; Schoppmann *et al*, 2003). Regarding tumour cell proliferation, Alani *et al* (2001) showed an inhibitory interaction between ID1 protein and the promoter region of the tumour suppressor gene *CDKN2A/p16*, supporting a role of ID1 as a potential oncogene. It was further shown that Id proteins also inhibit ETS transcription factors (Yates *et al*, 1999) which are able to influence p16 expression by binding to and activate its promoter (Ohtani *et al*, 2001). This indicates that Id proteins might inhibit the *p16* promoter, and thereby increase tumour cell proliferation, both directly and indirectly via interactions with ETS transcription factors.

Both Id proteins and ETS transcription factors have previously been implicated in the regulation of angiogenesis (Lyden *et al*, 1999; Wernert *et al*, 2003). Id proteins were shown to influence VEGF-dependent mobilisation of circulating endothelial cells and endothelial precursor cells from the bone marrow (Lyden *et al*, 2001). Further, ID1 might act by transcriptional repression of TSP-1, a well-known angiogenesis inhibitor (Volpert *et al*, 2002).

Regarding malignant melanoma, ID1 mRNA expression, assessed by *in situ* hybridisation, has been associated with loss of p16 protein in melanoma *in situ* (Polsky *et al*, 2001). In invasive melanomas, ID1 mRNA positivity was limited to the *in situ* component and perivascular tumour areas. These data might suggest a role of ID1 in regulating p16 expression in some early melanomas (Polsky *et al*, 2001). In our present study of nodular melanomas, which are considered to be more advanced primary tumours, we found no association between ID1 or ETS-1 transcription factor and p16 expression or angiogenic markers. On the other hand, strong ID1 expression was associated with thicker primary tumours and presence of *BRAF* mutations, as well as with significantly reduced patient survival, indicating an important role in melanoma progression. An inverse relation between ID1 and TSP-1 expression support a significant role of ID1 in the regulation of this complex and multitarget protein.

## MATERIALS AND METHODS

Of all melanomas occurring in Hordaland County (10% of the Norwegian population) during 1981–1997, 97.5% were diagnosed at The Gade Institute, Section for Pathology, Haukeland University Hospital. There were no differences in sex, anatomic site or stage between these cases and the 2.5% with a diagnosis from other laboratories, although the latter patients were 6 years younger (median age). The aim of this study was to focus on the aggressive subgroup consisting of nodular melanomas, which are all vertical growth phase (VGP) melanomas. After microscopic review of all cases diagnosed and recorded as malignant melanoma of the nodular type or not otherwise specified during this period, 202 cases were included. The presence of a VGP and the lack of a radial growth phase, that is, adjacent *in situ* or microinvasive component, were used as inclusion criteria (Elder and Murphy, 1991), and only primary tumours were included after careful examination of all slides. There was no history of familial occurrence. Complete information on patient survival, time and cause of death was available in all 202 cases. Last date of follow-up was December 18, 1998, and median follow-up time for all survivors was 76 months (range 13–210). Clinical follow-up (with respect to recurrences) was not carried out in 14 (mostly older) patients, and 21 patients were not treated with complete local excision. Thus, recurrence-free time could be studied in 167 patients.

### Immunohistochemistry

Immunohistochemistry (IHC) was performed on formalin-fixed and paraffin-embedded archival tissue. The technique of tissue microarray (TMA) was recently introduced (Kononen *et al*, 1998) and validated by independent studies of several tumour markers (Hoos *et al*, 2001; Nocito *et al*, 2001), and TMA slides were used for the staining of ID1 and ETS-1. For TMA construction (Kononen *et al*, 1998; Nocito *et al*, 2001), representative tumour areas were identified on H&E-stained slides. Tissue cylinders with a diameter of 0.6 mm were then punched from selected areas of the ‘donor’ block and mounted into a ‘recipient’ paraffin block using a custom-made precision instrument (Beecher Instruments, Silver Springs, MD, USA). As recommended (Hoos *et al*, 2001), three parallel tissue cylinders were sampled from each case, and these were taken from the suprabasal areas of the primary tumours. In some cases, a sufficient amount of tumour tissue was not available in the remaining paraffin blocks and 147 primary tumours and 56 metastases were available using the TMA technique. There was no significant difference regarding MVD or survival between the 147 cases included and those without sufficient material left for the TMA technique.

Sections (5 *μ*m) were dewaxed in xylene, and epitope retrieval was performed by microwaving for 3 × 5 min in Target Retrieval Solution pH 6.6 (TRS, Dako, Copenhagen, Denmark) at 500 W. The rabbit polyclonal antibody ID1 (SC-488, Santa Cruz Biotechnology Inc., Santa Cruz, CA, USA) was diluted 1 : 100 and incubated overnight at 4°C. Following microwaving for 3 × 5 min in 0.1 M Tris-HCl pH 9.0 with 2 mM EDTA, sections were incubated with the rabbit polyclonal antibody ETS-1 (SC-350, Santa Cruz Biotechnology Inc., Santa Cruz, CA, USA) diluted 1 : 500, for 30 min at room temperature. Immunoperoxidase staining was carried out using the Dako Envision Kit (Dako, Copenhagen, Denmark) and 3-amino-9-ethylcarbazole-peroxidase as substrate prior to counterstaining with Harris haematoxylin. To control for nonspecific staining, the primary antibodies were preincubated with specific blocking peptides (SC 488-P and SC 350-P, respectively), and this procedure completely blocked the staining signal in positive cases. Also, omission of the primary antibodies was used as a negative control. The specificity of both antibodies has been reported (Hollnagel *et al*, 1999; Nickoloff *et al*, 2000; Ohtani *et al*, 2001;Czuwara-Ladykowska *et al*, 2002; Chambers *et al*, 2003). The staining procedures and evaluation of other markers included have been described previously (Straume *et al*, 2000; Straume and Akslen, 2001, 2002), and the results of these are included for comparison (see Results).

### Evaluation of IHC

For all markers, both staining intensity and positive area were recorded (by one observer). A staining index (values 0–9), obtained as a product of staining intensity (0–3) and proportion of immunopositive tumour cells (⩽10%=1, 10–50%=2, >50%=3), was calculated as previously published (Aas *et al*, 1996; Straume and Akslen, 1997). For statistical purposes, cutoff points for continuous variables and staining index categories were based on the distribution curve for the values.

### Statistics

Analyses were performed using the statistical package SPSS ver. 10.1 (Norusis, 1994). Associations between different categorical variables were assessed by Pearson's *χ*^2^ test. Continuous variables not following the normal distribution were compared between two or more groups using the Mann–Whitney *U*-test or Kruskal–Wallis *H*-test. Univariate analyses of time to death due to malignant melanoma or time to recurrence (recurrence-free survival) were performed using the product-limit procedure (Kaplan–Meier method), with date of histologic diagnosis as the starting point. Patients who died of other causes were censored at the time of death. Differences between categories were tested by the log-rank test. The influence of covariates (Breslow's thickness, Clark's level of invasion, anatomic site, ulceration, vascular invasion, p53 protein expression, p16 protein expression, MVD and vascular invasion) on patient survival and recurrence-free survival was analysed by the proportional hazards method (Cox), including all variables with a *P*-value ⩽0.15 in univariate analyses, and tested by the likelihood ratio (lratio) test.

## RESULTS

### Expression of ID1 protein

**Figure 1:**
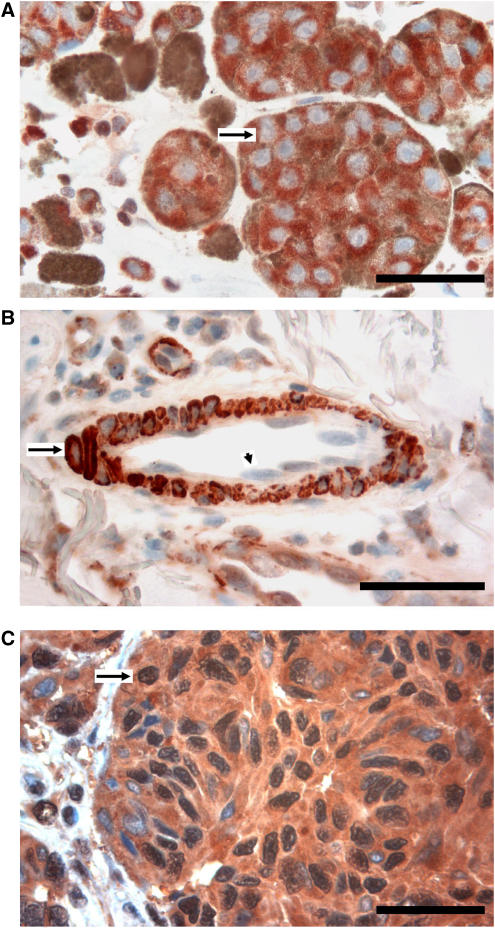
(**A**) Immunohistochemical staining of ID1 protein in human malignant melanoma. Note the strong cytoplasmic expression in tumour cells (arrow). Scale bar 50 *μ*m. (**B**) ID1 is strongly positive in perivascular cells (arrow), and some weaker staining is observed in the endothelial cells (arrowhead). (**C**) In this case, ETS-1 protein by immunohistochemistry is strongly positive in the cytoplasm and nuclei of tumour cells (arrow).

ID1 staining was seen in the cytoplasm in positive cases, whereas nuclear staining was observed only occasionally. Whereas, ID1 expression in stromal cells and keratinocytes was weak or negative, perivascular cells of intratumoural microvessels were strongly positive in most cases. Some weak ID1 expression was also observed in endothelial cells (Figure 1A and B). Normal melanocytes were negative for ID1 expression.

Only ID1 expression in tumour cells was quantified by the staining index. In total, 119 cases had sufficient tumour tissue in the TMA sections to be evaluated for ID1 staining, and only 8 cases were negative (staining index (SI)⩽1). In all, 61 cases had a staining index ⩾4 (median value), and were regarded to have strong immunohistochemical expression of ID1, whereas 58 cases had absent/weak expression (SI<4). Increased expression was significantly associated with increased tumour thickness (Table 1), and strong expression of ID1 was found significantly more frequent in primary melanomas located on the trunk, compared with other sites (*χ*^2^ test, *P*=0.001). Only a borderline association was found between ID1 staining and increased proliferation rate by Ki-67 expression (Table 1). No significant association was present between ID1 and loss of nuclear p16 protein expression, *p16* promoter hypermethylation or *p16* mutations. *BRAF* mutations have been found in 29% of these melanomas (Akslen *et al*, in press), and strong ID1 expression (SI⩾4) was significantly higher among these mutated cases, 71 *vs* 37% among the others (*χ*^2^ test, *P*=0.04). In contrast, NRAS mutations present (in 28% of the cases (Akslen *et al*, in press)) were significantly associated with decreased expression of the ID1-protein (*χ*^2^ test, *P*=0.034) (Table 1).

Strong ID1 expression was significantly associated with lower lymphatic vessel density, whereas no significant correlation was found with MVD (Table 1). We observed a significant association between increased ID1 and strong expression of both the tyrosine kinase receptor EPHA2 as well as its ligand Ephrin-A1 (*χ*^2^ test, *P*=0.048 and 0.001, respectively). Increased tumour cell staining of EPHA2 and Ephrin-A1 was found in 23 and 72%, respectively, in ID1 strong cases, compared with 9 and 47% in ID1 weak cases. Thrombospondin-1 expression in the tumour stroma was found in 34% of ID1 strong cases, compared with 57% in ID1 weak cases (*χ*^2^ test, *P*=0.014) (Table 1).

### Expression of ETS-1 protein

ETS-1 staining was predominantly seen in the cytoplasm of tumour cells in positive cases, although a mixed nuclear and cytoplasmic expression pattern was also observed (Figure 1C). Stromal cells, endothelial cells and perivascular cells, were most often negative, whereas scattered tumour-infiltrating lymphocytes were strongly positive in some cases.

A total of 125 cases had sufficient tumour tissue in the TMA sections to be evaluated for ETS-1 staining in tumour cells; and 26 cases (21%) were regarded to be negative (SI⩽1). In all, 55 cases had a staining index ⩾4, and were regarded to have strong immunohistochemical expression of ETS-1 whereas 70 cases had weak expression (SI<4). Cases with strong expression of ETS-1 had a significantly lower proliferative rate by Ki-67 (Mann–Whitney *U*-test, *P*=0.02). Median proliferative rate in cases with strong expression of ETS-1 was 23%, compared with 31% in cases with weak expression. There were no associations between ETS-1 staining and expression of p16 or ID1, nor with markers of angiogenesis or lymphangiogenesis.

### Survival analysis

**Figure 2:**
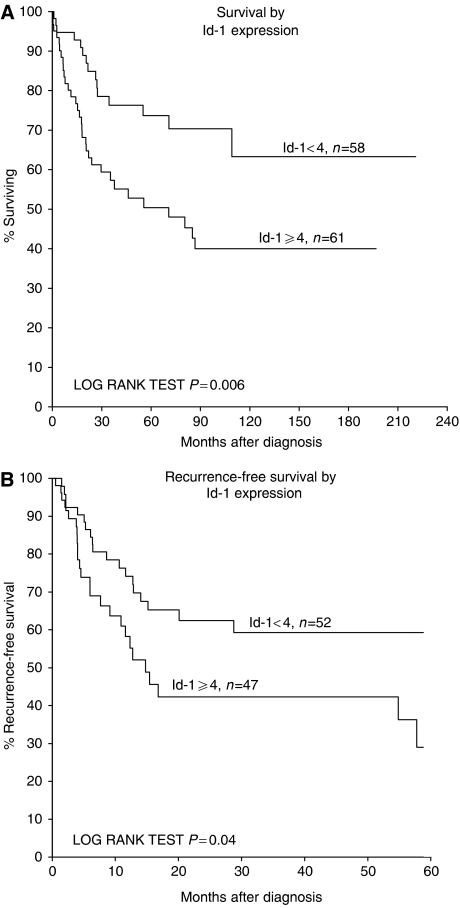
Survival curves according to the Kaplan–Meier method by ID1 expression in nodular melanomas. (**A**) Patient survival with death due to melanoma as end point. (**B**) Recurrence-free survival by ID1 expression.

As illustrated by the Kaplan–Meier plot in Figure 2, cases with increased expression of ID1 protein had significantly decreased patient survival in univariate survival analysis (log-rank, *P*=0.006). Also, recurrence-free survival was shorter in these cases (log-rank, *P*=0.04). Still, when adjusted for other strong prognostic factors in this patient series, like tumour thickness and others, ID1 expression had no independent prognostic significance in multivariate Cox analysis.

## DISCUSSION

In sporadic melanomas, various genetic alterations of *p16* have been reported, such as point mutations (0–26%), promoter methylation (0–19%), and homozygous deletions (6–25%)(Rocco and Sidransky, 2001; Straume *et al*, 2002). Still, no known genetic or epigenetic event can presently explain the lack of p16 expression in 45% of melanoma cases that we previously reported (Straume *et al*, 2000). Regulation of p16 expression has been suggested for the ID1 protein, both directly by inhibiting transcriptional activation at E-boxes within the *p16* promoter (Alani *et al*, 2001), or indirectly through ETS inhibition (Yates *et al*, 1999; Ohtani *et al*, 2001). In melanoma, a recent study suggested a role of ID1 in regulating *p16* expression in some early tumours (Polsky *et al*, 2001). In our present study of more advanced primary tumours, there was no significant association between ID1 or ETS-1 expression and p16 status, indicating that these regulatory pathways might not be central for *p16* inactivation in these particular tumours, and other mechanisms are likely to be involved at different stages of melanoma progression (Hara *et al*, 1996) (Bartek-Cancer res-96).

Still, strong ID1 expression indicated a more aggressive melanoma phenotype, and was found to be associated with increased tumour thickness, primary tumours located on the trunk, and a tendency towards increased tumour cell proliferation by Ki-67 expression. In addition, ID1 expression was significantly related to decreased recurrence-free and patient survival, and this has previously not been reported. The findings are in accordance with a few prognostic studies on other tumours. A study of breast cancer suggested that ID1 can control the malignant progression of breast cancer cells, particularly when mediated by sex steroid hormones (Lin *et al*, 2000). Another breast cancer study found a negative prognostic impact of increased ID1 expression (Schoppmann *et al*, 2003). Similar results have been published regarding pancreatic cancer (Lee *et al*, 2004), cervical cancer (Schindl *et al*, 2001), and ovarian carcinomas (Schindl *et al*, 2003). In these studies, stronger ID1 expression was consistently associated with poorly differentiated tumours and a more aggressive behaviour.

Loss of ETS-1 expression was significantly associated with increased tumour cell proliferation by Ki-67 expression, and this is consistent with a proposed role in cell cycle regulation, although probably not through the p16 pathway in melanomas. ETS-1 is a known downstream target of the RAS–RAF–MEK pathway regulating multiple cellular processes including proliferation (Graves and Petersen, 1998). Furthermore, a recent study has suggested that also ID1 expression and RAF/MEK activation might be related (Cheung *et al*, 2004). Ling *et al* (2002) suggested that proliferation in prostate cancer cells induced through activation of the Raf/Mek pathway might involve ID1. Notably, we found that ID1 expression was significantly increased in cases with *BRAF* mutations, indicating that ID1 could be a possible downstream target of this signalling pathway. The balance between ETS-1 or -2 and ID1 has been shown to influence cellular senescence through p16 protein expression, and this balance is also influenced by the RAS–RAF–MEK kinase cascade activity (Ohtani *et al*, 2001). The possible existence of a functional association between mutated *BRAF* and ID1 expression should be further investigated.

Similarly, a significant association between the RTK ligand and receptor pair ephrin-A1/EPHA2 and ID1 expression was suggested by our finding of a positive correlation with levels of ID1 protein. This association has previously not been reported. EPHA2 is involved in several signalling pathways and is reported to be overexpressed in many cancers, including melanoma (Nakamoto and Bergemann, 2002; Straume and Akslen, 2002).

ID1 has been implicated in the regulation of angiogenesis, and ID1/ID3-deficient mice revealed significant defects in vascularisation and extensive necrosis (Lyden *et al*, 2001). In this model, invasion of endothelial cells into nonvascular tissue, an important step in efficient tumour angiogenesis, was found to be inhibited, supporting a role of Id proteins as proangiogenic regulators. In a recent study of pancreatic cancer, increased ID1 expression was associated with increased MVD (Schoppmann *et al*, 2003). In an experimental study, thrombospondin-1 was transcriptionally repressed by ID1, and ID1 knockout mice showed suppressed angiogenesis and upregulated levels of TSP-1 (Volpert *et al*, 2002). In accordance with this, our results confirm the inverse relation between ID1 expression and TSP-1, although no significant association was found with MVD in these human melanomas.

In conclusion, our study suggests a significant role of ID1 protein in melanoma progression and survival. Increased ID1 expression was found in thicker tumours and was significantly associated with poor prognosis. No significant correlation was found with p16 alterations or MVD, suggesting that other regulatory pathways might be influenced by ID1 in these particular tumours. An inverse relation between ID1 and TSP-1 expression supports a significant role of ID1 in the regulation of this complex multitarget protein.

## Figures and Tables

**Table 1:** Immunohistochemical expression of ID1 in 119 vertical growth phase (nodular) melanomas in relation to markers of angiogenesis, lymphangiogenesis and tumour progression

**Variable**	**Absent/low ID1 expression^a^ (*n*=58)**	**High ID1 expression^b^ (*n*=61)**	***P*-value**
Median microvessel density (MVD)	119 mm^−2^	131 mm^−2^	0.6^c^
Median lymphatic vessel density (LVD)	12.5 mm^−2^	2.5 mm^−2^	**0.04** c
Median tumour thickness	3.5 mm	4.5 mm	**0.008** c
Median proliferative rate by Ki-67	27%	35%	0.08^c^
			
p16 expression present	30	32	NS
p16 expression absent	27	29	
			
BRAF mutations present	4	10	**0.044** d
BRAF mutations absent	15	9	
			
NRAS mutations present	11	2	**0.034** d
NRAS mutations absent	8	16	
			
Efrin-A1 expression strong	2	16	**0.001** d
Efrin-A1 expression weak	53	45	
			
EPHA2 expression strong	5	14	**0.048** d
EPHA2 expression weak	49	47	
			
TSP-1 expression strong	32	21	**0.014** d
TSP-1 expression weak	24	40	

NS=not significant.

a Staining index <4.

b Staining index ⩾4.

c Mann–Whitney *U*-test.

d Pearson's *χ*^2^ test.

Bold indicates significant values.
